# α-Lipoic Acid Alleviates Hepatic Lipid Deposition by Inhibiting FASN Expression via miR-3548 in Rats

**DOI:** 10.3390/nu13072331

**Published:** 2021-07-08

**Authors:** Shihui Guo, Kai Yan, Xi Fang, Yingdong Ni, Wenqiang Ma, Ruqian Zhao

**Affiliations:** 1 Key Laboratory of Animal Physiology and Biochemistry, Ministry of Agriculture and Rural Affairs, College of Veterinary Medicine, Nanjing Agricultural University, Nanjing 210095, China; 2017107012@njau.edu.cn (S.G.); 2016107015@njau.edu.cn (K.Y.); 2015107017@njau.edu.cn (X.F.); niyingdong@njau.edu.cn (Y.N.); zhao.ruqian@gmail.com (R.Z.); 2 MOE Joint International Research Laboratory of Animal Health & Food Safety, Nanjing Agricultural University, Nanjing 210095, China

**Keywords:** α-lipoic acid, hepatic lipid deposition, FASN, miRNA-3548, rat

## Abstract

Excessive liver lipid deposition is a vital risk factor for the development of many diseases. Here, we fed Sprague-Dawley rats with a control or α-lipoic acid-supplemented diet (0.2%) for 5 weeks to elucidate the effects of α-lipoic acid on preventive ability, hepatic lipid metabolism-related gene expression, and the involved regulatory mechanisms. In the current study, α-lipoic acid supplementation lowered plasma triglyceride level and hepatic triglyceride content. Reduced hepatic lipid deposition was closely associated with inhibiting fatty acid-binding protein 1 and fatty acid synthase expression, as well as increasing phosphorylated hormone-sensitive lipase expression at the protein level in α-lipoic acid-exposed rats. Hepatic miRNA sequencing revealed increased expression of miR-3548 targeting the 3′untranslated region of Fasn mRNA, and the direct regulatory link between miRNA-3548 and FASN was verified by dual-luciferase reporter assay. Taken together, α-lipoic acid lowered hepatic lipid accumulation, which involved changes in miRNA-mediated lipogenic genes.

## 1. Introduction

Excessive deposition of hepatic triglycerides has been associated with obesity [1], diabetes [2], hyperglycemia [3], and insulin resistance [4]. Numerous risk factors are thought to contribute to hepatic lipid accumulation, including diet, metabolic factors, genetics, and stress [5,6,7]. Recent data have revealed that the global prevalence of fatty liver disorders in the adult population is 24% [8]. Excessive hepatic lipid accumulation results in inflammation and liver cell damage and induces harmful effects, such as fibrosis and cirrhosis [9,10]. Several approved pharmacological agents have been applied to alleviate hepatic lipid deposition.

Hepatic lipid deposition is tightly controlled by key enzymes, including stearoyl- CoA desaturase (SCD), sterol acetyl CoA carboxylase (ACC), fatty acid synthase (FASN), carnitine palmitoyltransferase 1α (CPT1α), adipose triglyceride lipase (ATGL), hormone-sensitive lipase (HSL), cluster of differentiation 36 (CD36), and fatty acid-binding protein 1 (FABP1), which participate in the uptake, synthesis, transportation, and oxidation of fatty acids [11]. In particular, ACC, FASN, and SCD are responsible for lipogenesis [12,13], and ATGL, HSL, and CPT1α are key factors in lipolysis, while CD36 and FABP1 facilitate cellular uptake and intracellular trafficking of fatty acids [14]. Liver-specific knockout of CD36 or ACC1 reduces hepatic fat accumulation [15,16], while over-expression of CD36 increases hepatic fatty acid uptake and lipid deposition in vivo and in vitro [17]. HSL transgenic mice show increased hydrolytic activities against triacylglycerol and a diacylglycerol analog [18], whereas HSL-deficient mice present with decreased hormone-stimulated lipolysis [19]. Similarly, FABP1 ablation leads to a lower hepatic triglyceride content, supporting the role of FABP1 in the regulation of lipid disposal pathways [20,21]. These key regulators involved in hepatic lipid homeostasis play a crucial role in modulating hepatic concentrations of triglycerides.

α-Lipoic acid (LA) is a naturally occurring cofactor synthesized in most prokaryotic and eukaryotic microorganisms that acts as a strong antioxidant to repair oxidative damage by scavenging reactive oxygen species, chelating metal ions, and regenerating endogenous antioxidants [22]. α-Lipoic acid exerts various pharmacological activities for treating chronic diseases, such as Alzheimer’s disease, Down’s syndrome, diabetes mellitus, cognitive dysfunction, hypertension, and some cancers [23,24,25]. It also has anti-platelet activity and regulates muscle energy metabolism as well as other functions [26,27]. Many studies have suggested that α-lipoic acid suppresses lipid deposition in vivo and in vitro [28,29,30,31]. Park et al. (2008) revealed that α-lipoic acid markedly reduces plasma triglyceride levels and hepatic triglyceride content in high-fat-fed rats [28]. In addition to reduced hepatic lipid deposition, α-lipoic acid supplementation upregulates ATGL expression and downregulates FASN and phosphorylated ACC expression, which partially explains the lipid-lowering effect in mice treated with α-lipoic acid [30]. Interestingly, α-lipoic acid also reduces the accumulation of triglycerides by inhibiting lipogenesis through downregulation of FASN and SCD1 expression in human subcutaneous adipocytes [31]. Additionally, a supplement of 50 mg/kg α-lipoic acid reduces intramuscular triglycerides in the soleus muscle of obese Zucker rats [32]. However, an in-depth understanding of the regulatory mechanisms of α-lipoic acid on hepatic lipid deposition requires further exploration.

Therefore, the present study was performed to examine the changes in hepatic lipid metabolism-genes and proteins, as well as the expression of miRNAs targeting lipogenic genes in rats treated with α-lipoic acid, which will provide further insight into the lipid-lowering effect induced by α-lipoic acid.

## 2. Materials and Methods

### 2.1. Animals and Diets

Twenty-four male Sprague-Dawley rats (170–180 g) were randomized into the control group (CON) or the (±)-α-lipoic acid (Sigma-Aldrich, Shanghai, China) group (LA). These rats were given a chow diet or a chow diet plus 0.2% α-lipoic acid *ad libitum* for 5 weeks, respectively. The chow diet was prepared as described previously [33], the standard chow diet had 12.5% of energy derived from fat, 20.6% from protein, and 66.9% from carbohydrates. The rats were housed under standard environmental conditions at the laboratory animal center of Nanjing Agricultural University under constant temperature (20–26 °C), humidity (40–70%), and the natural photoperiod. Feed intake and weight were measured every 3 days during the experimental period. The rats were killed with 25% urethane anesthesia to obtain blood and liver samples at the end of the trial. Blood samples were collected in heparinized tubes and centrifuged at 900 RCF for 10 min to collect the plasma. The plasma and liver samples were stored at −80 °C until analysis. The animal handling and sampling procedures were consistent with the approved protocol of the Animal Ethics Committee of Nanjing Agricultural University. The sampling procedures complied with the Guidelines on Ethical Treatment of Experimental Animals (2006) No. 398 set by the Ministry of Science and Technology, China.

### 2.2. Serum and Hepatic Biochemical Analyses

Plasma cholesterol (CHOL), low-density lipoprotein cholesterol (LDL-C), high-density lipoprotein cholesterol (HDL-C) and total triglycerides (TGs) were detected using a biochemical automatic analyzer (Hitachi 7020, HITACHI, Tokyo, Japan) with the respective assay kits following the manufacturer’s instructions (ShinoTest Corp., Kanagawa, Tokyo, Japan). Hepatic CHOL and total TGs were extracted and measured using commercial assay kits (Applygen Technologies, Inc. Beijing, China).

### 2.3. Oil Red-O Staining

Oil Red-O staining was applied to assess lipid droplet formation in liver tissues according to a method described previously [34]. Briefly, fresh liver tissues were cut into small pieces and frozen immediately with dry ice, the liver tissue was immersed in OCT embedding agent, and was cut into 8-μm sections using a cryostat (Leica, CM3050S, Wentzler, Germany), and then the liver sections were incubated with 0.5 mg/mL of Oil Red-O staining solution for 5 min to show changes in fat accumulation. Hematoxylin staining was performed to visualize the cell nuclei. Finally, the sections were washed with running tap water and mounted with glycerin gelatin.

### 2.4. RNA Isolation and mRNA Quantification by Real-Time Polymerase Chain Reaction (PCR)

The detailed RNA isolation and mRNA quantification procedures were presented previously [35]. Briefly, TRIzol reagent (Invitrogen, Carlsbad, CA, USA) was used to isolate total RNA from 30 mg liver samples. Then, the quality and concentration of the RNA samples were checked with the NanoDrop-1000 spectrophotometer. All RNA samples were reverse-transcribed, diluted, and used as the template in PCR reactions with the real-time PCR system (Mx3000P, Stratagene, La Jolla, CA, USA). Glyceraldehyde-3-phosphate dehydrogenase was chosen as the reference gene for the liver. All primers used for this experiment are listed in Table S1. Data were analyzed by the 2^−ΔΔCt^ method.

### 2.5. Protein Extraction and Western Blot Analysis

Liver samples (approximately 50 mg) were mixed and homogenized with RIPA lysis buffer (150 mM NaCl, 1% Triton X-100, 50 mM pH 8.0 Tris-HCl, 0.1% sodium dodecyl sulfate, and 0.5% sodium deoxycholate) at a 1:100 ratio, then centrifuged at 4 °C and 13,800 RCF for 25 min. The supernatant was collected to evaluate protein content using a Pierce BCA Protein Assay kit (Thermo Scientific, Waltham, MA, USA). The supernatant sample (30 μg protein) was denatured, subjected to sodium dodecyl sulfate-polyacrylamide gel electrophoresis, transferred to a nitrocellulose membrane, incubated with primary and secondary antibodies, and applied to an enhanced chemiluminescence kit (Pierce Biotechnology Inc., Rockford, IL, USA). The details of the protein extraction and Western blot procedures have been shown previously [34]. Tubulin-α was selected as the loading control. The quantification and analysis of band intensity were performed with Quantity One software (Bio-Rad, Hercules, CA, USA) and GraphPad Prism 5.0 (GraphPad Software Inc., LA Jolla, CA, USA). See Table S2 for details of the primary and secondary antibodies.

### 2.6. MiRNA Sequencing, Bioinformatics Analyses, and In Vivo Validation

Briefly, sequencing libraries were generated using the NEBNext^®^ Multiplex Small RNA Library Prep Set for Illumina^®^ (NEB, Ipswich, MA, USA) according to the manufacturer’s recommendations. Library quality was assessed on the Agilent Bioanalyzer 2100 system using DNA High Sensitivity Chips (Novogene Co., Beijing, China). Then, purified hepatic RNAs (*n* = 3) were sequenced on an Illumina HiSeq 2500/2000 platform at Novogene Co. Sequences from 18–40 nt in length were used for further analysis. The *p*-values were adjusted using the Benjamini–Hochberg method. The hierarchical clustering heatmap was generated with ggplot library.

Based on the miRNA sequencing results, upregulated miRNAs of mature sequences from *Rattus norvegicus* were retrieved from the miRBase (http://www.mirbase.org/, October 2018) and applied to predict the binding capability of the target gene (FASN or FABP1) through complementary base pairing. The target miRNAs were verified in vivo using the miRNA reverse transcription kit and quantification kit (Hongsheng, Nanjing, China), as reported previously [36]. The sequences of all primers are listed in Table S3.

### 2.7. Cell Culture and Dual-Luciferase Reporter Assay

Approximately 1 × 105 HEK-293T cells/well were cultured in DMEM (Hyclone, Logan, UT, USA) containing 10% fetal bovine serum (Hyclone), 100 IU/mL penicillin, and 100 IU/mL streptomycin in a 24-well plate at 37 °C with 5% CO_2_. Following 24 h of culture, 1 μg miR-27a-3p, miR-3548, miR-182 mimics, and 50 ng pmirGLO-Fasn 3′UTR vector were co-transfected into cells with the jetPRIME transfection reagent (Hyclone). The dual-luciferase reporter analysis system (Promega, Madison, WI, USA) was used to detect firefly and Renilla luciferase signals 24 h after transfection according to the manufacturer’s instructions. Thus, the ratio between firefly luciferase activity and Renilla luciferase activity was relative luciferase activity.

Dual-luciferase reporter plasmids (pmirGLO-FASN 3′UTR vector and pmirGLO-FASN 3′UTR-MUT vector), miRNA mimics (miR-27a-3p, miR-3548, and miR-182), and the miRNA negative control (miR-NC) were constructed and synthesized by Hongsheng Biotechnology Co. Ltd. (Nanjing, China) as double-stranded 2′-O-methyl-modified RNA oligonucleotides.

### 2.8. Statistical Analysis

The data are presented as mean ± standard error mean and the differences between groups were analyzed using independent-samples *t* test with SPSS 20.0 software (SPSS Inc., Chicago, IL, USA). Graph Pad Prism 5.0 software was chosen to detect differences between the three groups using One-way ANOVA and Tukey’s test to compare data sets. *p*-values ≤ 0.05 were considered significant.

## 3. Results

### 3.1. α-Lipoic Acid Does Not Affect Rat Growth Performance

Supplementation with α-lipoic acid did not significantly affect the final weight, average daily gain, or average daily feed intake of rats during the 5-week feeding period (Table 1).

### 3.2. α-Lipoic Acid Lowers the Plasma Triglyceride Level and Hepatic Triglyceride Content

**Figure 1:**
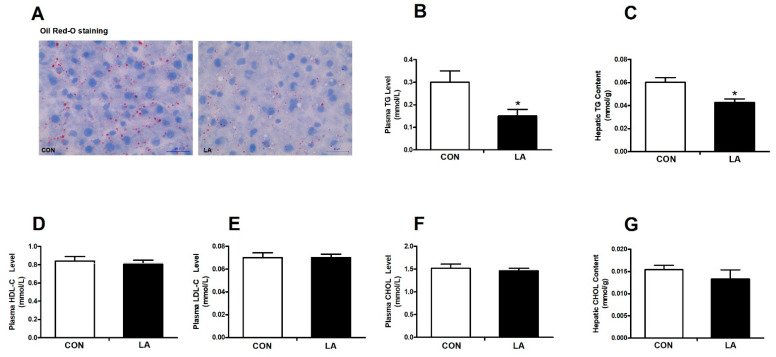
Effect of α-lipoic acid on lipid metabolism in rats. (**A**) Oil Red-O staining of a liver section (magnification, 400×). (**B**) Plasma triglyceride levels. (**C**) Hepatic triglyceride contents. (**D**) Plasma high-density lipoprotein levels. (**E**) Plasma low-density lipoprotein levels. (**F**) Plasma cholesterol levels. (**G**) Hepatic cholesterol contents. CON: Control diet, LA: Control diet+ alpha-lipoic acid. Values are expressed as mean ± SEM, *n* = 12 in each group, * *p* < 0.05.

As shown in Figure 1, the Oil Red-O staining results show that the LA group had fewer lipid droplets than the control group (Figure 1A). Correspondingly, adding dietary α-lipoic acid markedly decreased the plasma triglyceride level (Figure 1B) and hepatic triglyceride content (Figure 1C), but had no obvious effect on the plasma HDL-C level (Figure 1D), plasma LDL-C level (Figure 1E), plasma cholesterol level (Figure 1F), or hepatic cholesterol content (Figure 1G) in rats.

### 3.3. α-Lipoic Acid Reverses Hepatic Lipid Synthesis-Related Genes and Protein Expression

**Figure 2:**
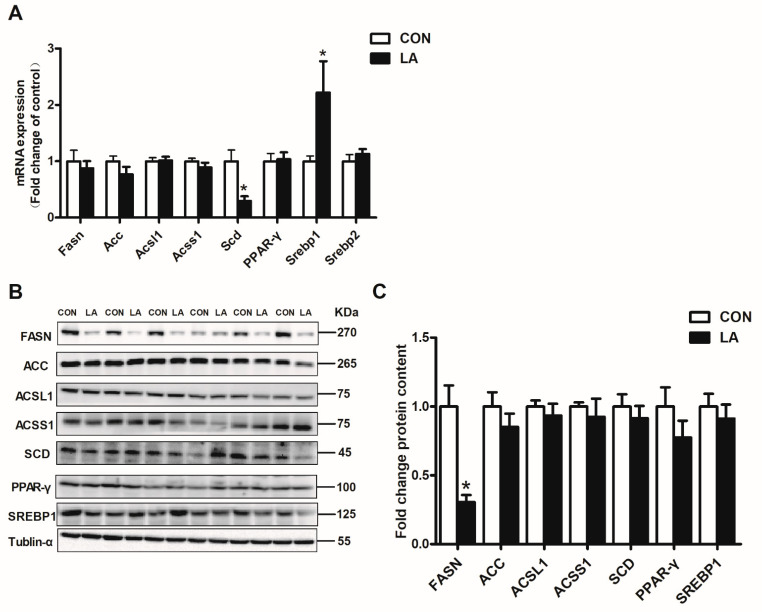
Relative hepatic lipid synthesis gene and protein expression. (**A**) Fasn, Acc, Acsl1, Acss1, Scd, Ppar-γ, Srebp1, and Srebp2 mRNA expression were evaluated by qRT-PCR. (**B**) Western blot analyses of FASN, ACC, ACSL1, ACSS1, SCD, PPAR-γ, SREBP1, and tubulin-α proteins. (**C**) Densitometric analysis of FASN, ACC, ACSL1, ACSS1, SCD, PPAR-γ, and SREBP1 protein levels. CON: Control diet, LA: Control diet+ alpha-lipoic acid. Values are expressed as mean ± SEM, *n* = 6 in each group, * *p* < 0.05.

Adding α-lipoic acid to the diet decreased hepatic Scd expression, but increased Srebp1 expression at the mRNA level in the rats (Figure 2A). In addition, supplementing with α-lipoic acid did not affect the hepatic Fasn mRNA level, but downregulated hepatic FASN expression at the protein level in rats (Figure 2B,C).

### 3.4. α-Lipoic Acid Reverses Hepatic Lipolysis and Fatty Acid Transport-Related Gene and Protein Expression

**Figure 3:**
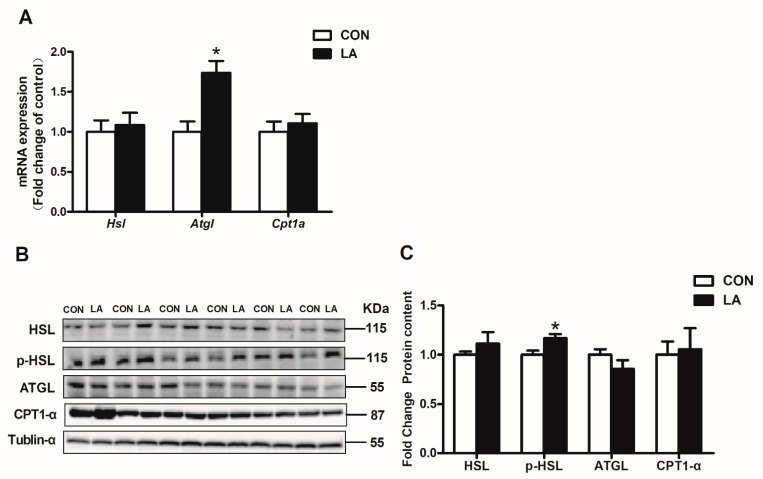
Relative hepatic lipolysis gene and protein expression. (**A**) qRT-PCR analysis of Hsl, Atgl, and Cpt1α mRNA expression. (**B**) HSL, p-HSL, ATGL, CPT1α, and tubulin-α protein levels were evaluated by Western blotting. (**C**) Densitometric analysis of HSL, p-HSL, ATGL, and CPT1-α protein expression. CON: Control diet, LA: Control diet+ alpha-lipoic acid.Values are expressed as mean ± SEM, *n* = 6 in each group, * *p* < 0.05.

**Figure 4:**
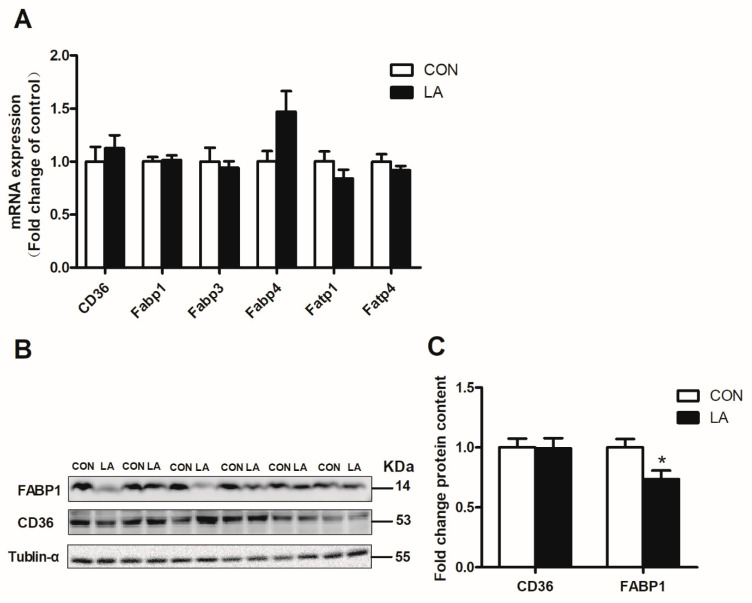
Relative hepatic lipid transport gene and protein expression. (**A**) qRT-PCR analysis of CD36, Fabp1, Fabp3, Fabp4, Fatp1, and Fatp4 mRNA expression. (**B**) FABP1, CD36, and tubulin-α protein levels were evaluated by Western blotting. (**C**) Densitometric analysis of FABP1 and CD36 protein expression. CON: Control diet, LA: Control diet+ alpha-lipoic acid. Values are expressed as mean ± SEM, *n* = 6 in each group, * *p* < 0.05.

The majority of genes involved in lipolysis and fatty acid transport remained unchanged, whereas Atgl mRNA level increased significantly in the livers of α-lipoic acid-fed rats (Figure 3A and Figure 4A). The α-lipoic acid supplement markedly enhanced hepatic p-HSL protein expression (Figure 3B,C), but downregulated hepatic FABP1 protein expression (Figure 4B,C) in the rats.

### 3.5. α-Lipoic Acid Downregulates FASN Expression through miRNA-3548

**Figure 5:**
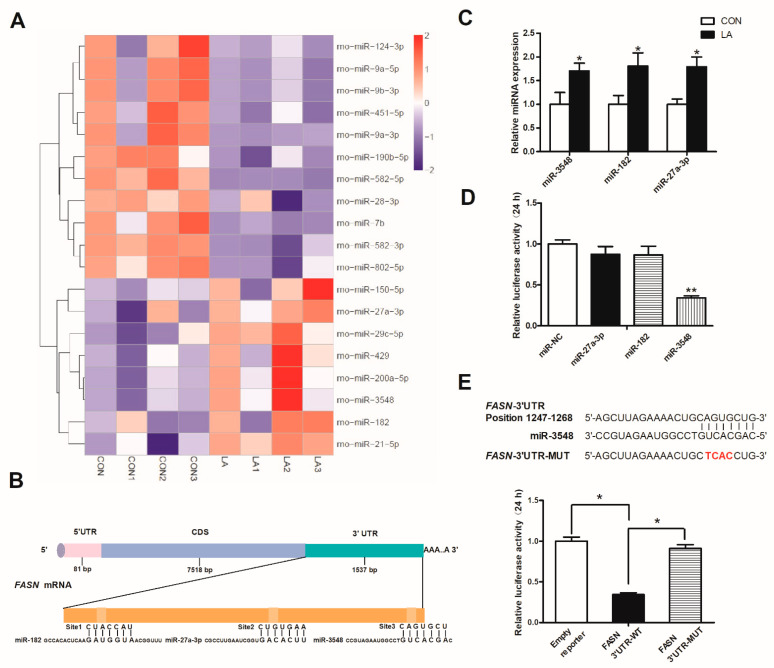
α-Lipoic acid downregulates FASN expression through miRNA-3548 in the liver. (**A**) Heatmap of miRNA expression profiles based on miRNA sequencing. (**B**) Predicted binding sites of miR-27a-3p, miR-3548, and miR-182 in the 3′UTR of Fasn mRNA. (**C**) qRT-PCR analysis of hepatic miR-3548, miR-182, and miR-27a-3p expression. (**D**) Relative luciferase activity was detected when HEK-293T cells were co-transfected with the Fasn 3′UTR reporter and NC, miR-27a-3p, miR-182, or miR-3548 mimics for 24 h, the global ANOVA *p*-value < 0.01. (**E**) HEK-293T cells were co-transfected with miR-3548 and either the empty vector, wild-type, or binding site mutant-reporter and luciferase activity was measured 24 h post-transfection, the global ANOVA *p*-value < 0.01. CON: Control diet, LA: Control diet+ alpha-lipoic acid. Values are expressed as mean ± SEM, *n* = 6 in each group, * *p* < 0.05, ** *p* < 0.01.

The heatmap of the miRNA expression profiles revealed eight upregulated and 11 downregulated hepatic miRNAs between the two groups (Figure 5A and Figure S1). These results show that α-lipoic acid affects FASN, p-HSL, and FABP1 protein expression. We used software to analyze and predict whether the 19 changed miRNAs had binding sites with the three different proteins. Then, miR-182, miR-27a-3p, and miR-3548 were predicted to bind to the 3′UTR of the Fasn gene (Figure 5B), which was verified in the liver of rats in response to feeding α-lipoic acid (Figure 5C). We co-transfected the three miRNA mimics and the 3′UTR region of FASN into 293T cells. Co-transfection of the pmirGLO-Fasn 3′UTR vector with the miR-3548 mimic led to a 60% decrease in luciferase activity compared with the control group, but the other two miRNA mimics did not have similar effects (Figure 5D). Rescued luciferase activity was detected when miR-3548-binding sites in the 3′UTR of Fasn mRNA were mutated (Figure 5E). These results indicate that FASN is a direct target of miR-3548.

## 4. Discussion

α-Lipoic acid has been reported to decrease body weight, inhibit feed intake, and reduce lipid deposition in rodents and broilers [28,29,37,38,39,40]. Shen et al. (2005) observed a reduction in body weight and feed consumption in mice fed a normal diet containing 0.5% or 1% α-lipoic acid during a 3-week trial [37]. Zhang et al. (2008) demonstrated that α-lipoic acid inhibits weight gain in mice fed 0.2% α-lipoic acid and high fat (15% or 4%) diet [38]. Furthermore, supplementation with 0.25, 0.5%, or 1% α-lipoic acid moderately reduces body weight and feed intake of high fat-fed rats [28,29,39]. Moreover, α-lipoic acid exhibits an anti-adiposity effect, as represented by lower body weight and fat mass in overweight/obese humans [41,42,43]. Therefore, inhibiting α-lipoic acid-induced growth is closely related to decreased feed intake [30] and lowering lipid deposition by disrupting lipogenesis and lipolysis [31]. Here, our results demonstrate that 0.2% α-lipoic acid had no effect on body weight or feed intake in rats fed a chow diet during a 5-week period, which was different from the results reported by others. These findings indicate that the magnitude of weight change induced by α-lipoic acid is probably attributed to a dose-response.

Numerous studies have demonstrated a distinct link between α-lipoic acid and liver lipid-lowering effects [28,29,30,31]. Our data confirm the lipid-lowering effect induced by α-lipoic acid, as shown by a reduction in plasma triglyceride levels and hepatic triglyceride contents in rats. Moreover, α-lipoic acid significantly downregulated FASN and FABP1 protein expression but upregulated p-HSL protein expression in the liver of rats. Previous studies have suggested that FASN synthesizes long-chain fatty acids by catalyzing acetyl-CoA and malonyl-CoA [44]. SCD synthesizes monounsaturated fatty acids by introducing hydrogen bonds between the 9th and 10th carbon atoms [45]. Another report showed that α-lipoic acid supplementation suppresses *de novo* lipogenesis by reducing FASN and SCD1 in mice [30,31]. Recent results support α-lipoic acid as an effective mitochondrial nutrient to improve insulin resistance in Htd2 knockdown adipocytes [46]. Furthermore, FABP1-ablated mice show reduced hepatic TGs through modulation of murine stellate cell activation or disruption of net fatty acid uptake and utilization [47,48]. In addition, HSL is one of the key rate-limiting enzymes in lipolysis [49], whereas PKA-mediated phosphorylation of Ser552, Ser649, and Ser650 on HSL promotes activation of lipase by increasing enzyme activity two-fold [50]. The reduction in hepatic lipid induced by α-lipoic acid is probably due to downregulated expression of proteins involved in lipogenesis and upregulated expression of proteins involved in lipolysis.

Disruption of liver lipid homeostasis occurs when the rate of hepatic fatty acid uptake from plasma and de novo fatty acid synthesis is greater than the rate of fatty acid oxidation and export [51]. Therefore, the regulation of hepatic lipid deposition induced by α-lipoic acid is a genetic modulation of lipogenesis, mainly through the downregulation of FASN expression. We observed an unchanged Fasn mRNA level and reduced FASN protein expression level in the liver of the α-lipoic acid-fed rats, suggesting the possible involvement of post-transcriptional regulation of the FASN gene.

MiRNAs participate predominantly in post-transcriptional regulation through binding to complementary sites in the 3′UTR of target mRNAs and drive translational repression or mRNA degradation [52]. Song et al. revealed that miR-195 exerts its essential tumor-suppressive role by targeting FASN during the progression of malignant meningioma [53]. Moreover, obesity-associated fatty liver can be alleviated by upregulated expression of miR-103 via downregulating Fasn and Scd1 in db/db mice [54]. In the current study, miRNA sequencing presented different miRNA expression profiles, revealing 11 downregulated miRNAs and eight upregulated miRNAs in the liver of the α-lipoic acid-fed rats. Three of eight upregulated miRNAs were predicted to target the Fasn gene, but only miR-3548 was confirmed to directly target the 3′UTR of Fasn mRNA according to the dual-luciferase reporter assay result [55,56]. Therefore, miRNA sequencing and dual-luciferase reporter assay were conducted to confirm that the increase in hepatic miR-3548 directly targeted the 3′UTR of Fasn mRNA in the α-lipoic acid-fed rats, indicating miRNA-mediated translation repression of FASN.

## 5. Conclusions

**Figure 6:**
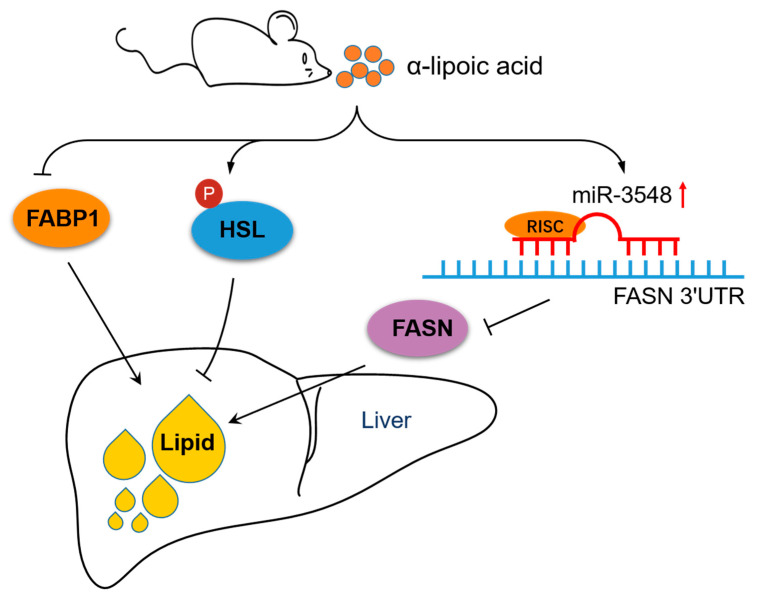
Schematic diagram of the mechanisms of α-lipoic acid related to reduced hepatic lipid accumulation. α-Lipoic acid suppresses FASN and FABP1 expression and enhances p-HSL expression, resulting in reduced hepatic lipid deposition. Impaired FASN expression is mediated by miR-3548 in the liver of α-lipoic acid-fed rats. FABP1: Fatty acid-binding protein 1, HSL: Hormone-sensitive lipase, FASN: Fatty acid synthase, RISC: RNA-induced silencing complex.

Taken together, α-lipoic acid alleviated hepatic lipid deposition by downregulating FASN and FABP1 protein expression and upregulating p-HSL expression at the protein level. Increasing miR-3548 level plays a role in inhibiting hepatic FASN expression of rats, which provides a new target for regulating lipid biosynthesis. A schematic description proposed by our data is shown in Figure 6.

## Figures and Tables

**Table 1:** Effect of α-lipoic acid on rat growth performance.

Parameters	CON	LA	*p*-Value
Initial body weight (g)	179.07 ± 3.37	175.33 ± 2.01	0.43
Final body weight (g)	416.51 ± 12.68	401.86 ± 9.51	0.49
ADG (g/d)	6.99 ± 0.32	6.66 ± 0.24	0.43
ADFI (g/d)	24.84 ± 0.86	23.55 ± 0.63	0.16

Note: CON: Control diet, LA: Control diet+ alpha-lipoic acid. ADG, average daily gain; ADFI, average daily feed intake. Values are expressed as mean ± SEM, *n* = 12 in each group, *p* < 0.05 indicates a significant difference between the two groups.

## Data Availability

Data is contained within the article or Supplementary Material.

## Supplementary Materials

The following are available online at https://www.mdpi.com/article/10.3390/nu13072331/s1, Table S1: Nucleotide sequences of specific primers, Table S2: Details of antibodies used in the experiment, Table S3: miRNA and the corresponding primer sequences, Figure S1: Volcano plot depicting differential expression of hepatic miRNAs in rats fed on α-lipoic acid.

## References

[1] Ibrahim S.H., Kohli R., Gores G.J. (2011). Mechanisms of lipotoxicity in NAFLD and clinical implications. J. Pediatr. Gastr. Nutr..

[2] Targher G., Bertolini L., Padovani R., Rodella S., Tessari R., Zenari L., Day C., Arcaro G. (2007). Prevalence of nonalcoholic fatty liver disease and its association with cardiovascular disease among type 2 diabetic patients. Diabetes Care.

[3] Fan H., Ma X., Lin P., Kang Q., Zhao Z., Wang L., Sun D., Cheng J., Li Y. (2017). Scutellarin prevents nonalcoholic fatty liver disease (NAFLD) and hyperlipidemia via PI3K/AKT-dependent activation of nuclear factor (Erythroid-derived 2)-like 2 (Nrf2) in rats. Med. Sci. Monit..

[4] Kitade H., Chen G., Ni Y., Ota T. (2017). Nonalcoholic fatty liver disease and insulin resistance: New insights and potential new treatments. Nutrients.

[5] Ullah R., Rauf N., Nabi G., Ullah H., Shen Y., Zhou Y.D., Fu J. (2019). Role of nutrition in the pathogenesis and prevention of non-alcoholic fatty liver disease: Recent updates. Int. J. Biol. Sci..

[6] Haslam D.W., James W.P. (2005). Obesity. Lancet.

[7] Wright S.M., Aronne L.J. (2012). Causes of obesity. Abdom. Imaging.

[8] Younossi Z., Anstee Q.M., Marietti M., Hardy T., Henry L., Eslam M., George J., Bugianesi E. (2018). Global burden of NAFLD and NASH: Trends, predictions, risk factors and prevention. Nat. Rev. Gastroenterol. Hepatol..

[9] Bessone F., Razori M.V., Roma M.G. (2019). Molecular pathways of nonalcoholic fatty liver disease development and progression. Cell. Mol. Life Sci..

[10] Pierantonelli I., Svegliati-Baroni G. (2019). Nonalcoholic fatty liver disease: Basic pathogenetic mechanisms in the progression from NAFLD to NASH. Transplantation.

[11] Ipsen D.H., Lykkesfeldt J., Tveden-Nyborg P. (2018). Molecular mechanisms of hepatic lipid accumulation in non-alcoholic fatty liver disease. Cell. Mol. Life Sci..

[12] Ameer F., Scandiuzzi L., Hasnain S., Kalbacher H., Zaidi N. (2014). De novo lipogenesis in health and disease. Metabolism.

[13] Solinas G., Boren J., Dulloo A.G. (2015). De novo lipogenesis in metabolic homeostasis: More friend than foe?. Mol. Metab..

[14] Glatz J.F., van der Vusse G.J. (1996). Cellular fatty acid-binding proteins: Their function and physiological significance. Prog. Lipid Res..

[15] Mao J., DeMayo F.J., Li H., Abu-Elheiga L., Gu Z., Shaikenov T.E., Kordari P., Chirala S.S., Heird W.C., Wakil S.J. (2006). Liver-specific deletion of acetyl-CoA carboxylase 1 reduces hepatic triglyceride accumulation without affecting glucose homeostasis. Proc. Natl. Acad. Sci. USA.

[16] Wilson C., Tran J.L., Erion D.M., Vera N.B., Febbraio M., Weiss E.J. (2016). Hepatocyte-specific disruption of CD36 attenuates fatty liver and improves insulin sensitivity in HFD-fed mice. Endocrinology.

[17] Koonen D.P., Jacobs R.L., Febbraio M., Young M.E., Soltys C.L., Ong H., Vance D.E., Dyck J.R. (2007). Increased hepatic CD36 expression contributes to dyslipidemia associated with diet-induced obesity. Diabetes.

[18] Lucas S., Tavernier G., Tiraby C., Mairal A., Langin D. (2003). Expression of human hormone-sensitive lipase in white adipose tissue of transgenic mice increases lipase activity but does not enhance in vitro lipolysis. J. Lipid Res..

[19] Haemmerle G., Zimmermann R., Hayn M., Theussl C., Waeg G., Wagner E. (2002). Hormone-sensitive lipase deficiency in mice causes diglyceride accumulation in adipose tissue, muscle, and testis. J. Biol. Chem..

[20] Newberry E.P., Xie Y., Kennedy S., Han X., Buhman K.K., Luo J., Gross R.W., Davidson N.O. (2003). Decreased hepatic triglyceride accumulation and altered fatty acid uptake in mice with deletion of the liver fatty acid-binding protein gene. J. Biol. Chem..

[21] Martin G.G., Atshaves B.P., Huang H., McIntosh A.L., Williams B.J., Pai P.J. (2009). Hepatic phenotype of liver fatty acid binding protein gene-ablated mice. Am. J. Physiol. Gastrointest. Liver Physiol..

[22] Moura F.A., de Andrade K.Q., dos Santos J.C., Goulart M.O. (2015). Lipoic acid: Its antioxidant and anti-inflammatory role and clinical applications. Curr. Top. Med. Chem..

[23] Biewenga G.P., Haenen G.R., Bast A. (1997). The pharmacology of the antioxidant lipoic acid. Gen. Pharmacol..

[24] Gomes M.B., Negrato C.A. (2014). Alpha-lipoic acid as a pleiotropic compound with potential therapeutic use in diabetes and other chronic diseases. Diabetol. Metab. Syndr..

[25] Zhang Y.H., Wang D.W., Xu S.F., Zhang S., Fan Y.G., Yang Y.Y., Guo S.Q., Wang S., Guo T., Wang Z.Y. (2018). Alpha-Lipoic acid improves abnormal behavior by mitigation of oxidative stress, inflammation, ferroptosis, and tauopathy in P301S Tau transgenic mice. Redox Biol..

[26] Lai Y.S., Shih C.Y., Huang Y.F., Chou T.C. (2010). Antiplatelet activity of alpha-lipoic acid. J. Agric. Food Chem..

[27] Jing Y., Cai X., Xu Y., Zhu C., Wang L., Wang S., Zhu X., Gao P., Zhang Y., Jiang Q. (2016). Alpha-Lipoic acids promote the protein synthesis of C2C12 myotubes by the TLR2/PI3K signaling pathway. J. Agric. Food Chem..

[28] Park K.G., Min A.K., Koh E.H., Kim H.S., Kim M.O., Park H.S., Kim Y.D., Yoon T.S., Jang B.K., Hwang J.S. (2008). Alpha-lipoic acid decreases hepatic lipogenesis through adenosine monophosphate-activated protein kinase (AMPK)-dependent and AMPK-independent pathways. Hepatology.

[29] Valdecantos M.P., Perez-Matute P., Gonzalez-Muniesa P., Prieto-Hontoria P.L., Moreno-Aliaga M.J., Martinez J.A. (2012). Lipoic acid administration prevents nonalcoholic steatosis linked to long-term high-fat feeding by modulating mitochondrial function. J. Nutr. Biochem..

[30] Yang Y., Li W., Liu Y., Sun Y., Li Y., Yao Q., Li J., Zhang Q., Gao Y., Gao L. (2014). Alpha-lipoic acid improves high-fat diet-induced hepatic steatosis by modulating the transcription factors SREBP-1, FoxO1 and Nrf2 via the SIRT1/LKB1/AMPK pathway. J. Nutr. Biochem..

[31] Fernandez-Galilea M., Perez-Matute P., Prieto-Hontoria P.L., Sainz N., Lopez-Yoldi M., Houssier M., Martínez J.A., Langin D., Moreno-Aliaga M.J. (2014). Alpha-lipoic acid reduces fatty acid esterification and lipogenesis in adipocytes from overweight/obese subjects. Obesity.

[32] Teachey M.K., Taylor Z.C., Maier T., Saengsirisuwan V., Sloniger J.A., Jacob S., Klatt M.J., Ptock A., Kraemer K., Hasselwander O. (2003). Interactions of conjugated linoleic acid and lipoic acid on insulin action in the obese Zucker rat. Metabolism.

[33] Guo S., Yang C., Jiang S., Ni Y., Zhao R., Ma W. (2020). Repeated Restraint Stress Enhances Hepatic TFR2 Expression and Induces Hepatic Iron Accumulation in Rats. Biol. Trace Elem. Res..

[34] Mehlem A., Hagberg C.E., Muhl L., Eriksson U., Falkevall A. (2013). Imaging of neutral lipids by oil red O for analyzing the metabolic status in health and disease. Nat. Protoc..

[35] Jiang S., Guo S., Li H., Ni Y., Ma W., Zhao R. (2019). Identification and functional verification of microRNA-16 family targetingintestinal divalent metal transporter 1 (DMT1) In Vitro and In Vivo. Front. Physiol..

[36] Jiang S., Fang X., Liu M., Ni Y., Ma W., Zhao R. (2019). MiR-20b down-regulates intestinal ferroportin expression In Vitro and In Vivo. Cells.

[37] Shen Q.W., Jones C.S., Kalchayanand N., Zhu M.J., Du M. (2005). Effect of dietary alpha-lipoic acid on growth, body composition, muscle pH, and AMP-activated protein kinase phosphorylation in mice. J. Anim. Sci..

[38] Zhang W.J., Bird K.E., McMillen T.S., LeBoeuf R.C., Hagen T.M., Frei B. (2008). Dietary alpha-lipoic acid supplementation inhibits atherosclerotic lesion development in apolipoprotein E-deficient and apolipoprotein E/low-density lipoprotein receptor-deficient mice. Circulation.

[39] Seo E.Y., Ha A.W., Kim W.K. (2012). Alpha-Lipoic acid reduced weight gain and improved the lipid profile in rats fed with high fat diet. Nutr. Res. Pract..

[40] Arshad M.S., Anjum F.M., Khan M.I., Shahid M. (2013). Wheat germ oil and alpha-lipoic acid predominantly improve the lipid profile of broiler meat. J. Agric. Food Chem..

[41] Carbonelli M.G., Di Renzo L., Bigioni M., Di Daniele N., De Lorenzo A., Fusco M.A. (2010). Alpha-lipoic acid supplementation: A tool for obesity therapy?. Curr. Pharm. Des..

[42] Kim E., Park D.W., Choi S.H., Kim J.J., Cho H.S. (2008). A preliminary investigation of alpha-lipoic acid treatment of antipsychotic drug-induced weight gain in patients with schizophrenia. J. Clin. Psychopharmacol..

[43] Li N., Yan W., Hu X., Huang Y., Wang F., Zhang W., Wang Q., Wang X., Sun W. (2017). Effects of oral alpha-lipoic acid administration on body weight in overweight or obese subjects: A crossover randomized, double-blind, placebo-controlled trial. Clin. Endocrinol..

[44] Jayakumar A., Chirala S.S., Chinault A.C., Baldini A., Abu-Elheiga L., Wakil S.J. (1994). Isolation and chromosomal mapping of genomic clones encoding the human fatty acid synthase gene. Genomics.

[45] Ducheix S., Peres C., Hardfeldt J., Frau C., Mocciaro G., Piccinin E., Lobaccaro J.M., De Santis S., Chieppa M., Bertrand-Michel J. (2018). Deletion of stearoyl-coA desaturase-1 from the intestinal epithelium promotes inflammation and tumorigenesis, reversed by dietary oleate. Gastroenterology.

[46] Zhen M., Xu J., Zhang Z., Zou X., Wang X., Wang X. (2021). Htd2 deficiency-associated suppression of α-lipoic acid production provokes mitochondrial dysfunction and insulin resistance in adipocytes. Redox Biol..

[47] Chen A., Tang Y., Davis V., Hsu F.F., Kennedy S.M., Song H., Turk J., Brunt E.M., Newberry E.P., Davidson N.O. (2013). L-FABP modulates murine stellate cell activation and diet induced nonalcoholic fatty liver disease. Hepatology.

[48] Newberry E.P., Kennedy S.M., Xie Y., Sternard B.T., Luo J., Davidson N.O. (2008). Diet-induced obesity and hepatic steatosis in L-FABP-/- mice is abrogated with SF, but not PUFA, feeding and attenuated after cholesterol supplementation. Am. J. Physiol. Gastrointest. Liver Phys..

[49] Duncan R.E., Ahmadian M., Jaworski K., Sarkadi-Nagy E., Sul H.S. (2007). Regulation of lipolysis in adipocytes. Annu. Rev. Nutr..

[50] Nielsen T.S., Jessen N., Jorgensen J.O., Moller N., Lund S. (2014). Dissecting adipose tissue lipolysis: Molecular regulation and implications for metabolic disease. J. Mol. Endocrinol..

[51] Fabbrini E., Sullivan S., Klein S. (2010). Obesity and nonalcoholic fatty liver disease: Biochemical, metabolic, and clinical implications. Hepatology.

[52] Bartel D.P. (2004). MicroRNAs: Genomics, biogenesis, mechanism, and function. Cell.

[53] Song L.R., Li D., Weng J.C., Li C.B., Wang L., Wu Z., Zhang J.T. (2020). MicroRNA-195 functions as a tumor suppressor by directly targeting fatty acid synthase in malignant meningioma. World Neurosurg..

[54] Zhang M., Tang Y., Tang E., Lu W. (2020). MicroRNA-103 represses hepatic de novo lipogenesis and alleviates NAFLD via targeting FASN and SCD1. Biochem. Biophys. Res. Commun..

[55] Yang Z., Zhong L., Xian R., Yuan B. (2015). MicroRNA-223 regulates inflammation and brain injury via feedback to NLRP3 inflammasome after intracerebral hemorrhage. Mol. Immunol..

[56] Jiang S., Yan K., Sun B., Gao S., Yang X., Ni Y., Ma W., Zhao R. (2018). Long-Term high-fat diet decreases hepatic iron storage associated with suppressing TFR2 and ZIP14 expression in rats. J. Agric. Food Chem..

