# Supplementary material for: α-Lipoic Acid Alleviates Hepatic Lipid Deposition by Inhibiting FASN Expression via miR-3548 in Rats
Source: Nutrients. 2021 Jul 8;13(7):2331. doi: 10.3390/nu13072331 (PMC8308747; doi:10.3390/nu13072331)
Supplement: Supplementary file 1 [file nutrients-13-02331-s001.zip › nutrients-1247983-supplementary.pdf]

Supplementary Table S1. Nucleotide sequences of specific primers

Target genes	Sequences (5’ to 3’)	GenBank No.
Fasn	F: CTCCTTAGTAGTGCGTGGTC<br>R:ATGCTGCTCAAACGATGT	NM_017332.1
Acc	F: GCTTCTGCGACTCCCC<br>R:TGAAATCCTTTTGTGCAACTA	NM_022193.1
Scd	F: TCGTCAGCACCTTCTTGAGATA<br>R:CAGCCATGCAGTCGATGAAG	NM_139192.2
Acsl1	F: AGGAAGGATGCTGTGTAG<br>R:TGTCAAGAGG AGGCTGTT	NM_012820.1
Acss1	F: ATCAGACT CCAGCATCAAC<br>R: CCAATACACAGGTCACAGA	NM_001106524.1
Srebp1	F: CACTTACGGTCAGCACTT<br>R: CACAACTCACTGGACTTAGA	NM_001276707.1
Srebp2	F: GTTGGTGGTTGGTGAAGA<br>R: ACATAGTGCGTGTCAGTC	NM_001033694.1
Ppar-γ	F: GCCGTTTCCACAAGTGCCT<br>R:CTTTCCTGCGAGTATGACCC	NM_013196.1
Hsl	F: GAAGGCAAGATGGCACTA<br>R: TTACAGCAGAAGAGGCATAT	NM_012859.1
Atgl	F: TAGAGGGTGTCCGCAATGTG<br>R:CAGTGGGAAGGTGTAGTCAGTC	NM_001046005.2
Cpt1α	F: CTTATCGTGGTGGTGGGTGT<br>R:TCATTTTGCCGTGTTCTGC	NM_031559.2
Fabp1	F: GTCACTACTGGAACTTCACA<br>R: TTCAGGTAATGCGTCTCAAT	NM_012556.2
Fabp3	F: GCAGAAGCCATCTCAAGA	NM_024162.1

	R: TTCCAAGAAGTCACAGAGG	
Fabp4	F: AGGAAGGATGCTGTGGAT<br>R: TGTCAAGAGGAGGCTGTT	NM_053365.1
CD36	F: TTGCCTCCCCACTCCAGAA<br>R:TGGCTTGACCAGTATGTTGACCT	NM_031561.2
FATP1	F: CACTTACGGTCAGCACTT<br>R: CACAACTCACTGGACTTAGA	NM_031736.1
FATP4	F: GTTGGTGGTTGGTGAAGA<br>R: ACATAGTGCGTGTCAGTC	NM_001100706.1
GAPDH	F: AACAGGGTGGTGGACCTCAT<br>R:GATGGTATTCGAGAGAAGGGAGG	NM_017008.4

Supplementary Table S2. Details of antibodies used in the experiment

Antibodies	Source	Catalogue no.	Dilution ratio
Primary antibody			
FASN	Abcam	AB43451	1:1000
ACC	Bioworld	BS1377	1:1000
ACSL1	Santa Cruz	sc-98925	1:1000
ACSS1	Santa Cruz	sc-373847	1:1000
SCD	Santa Cruz	sc-30081	1:200
SREBP1	Proteintech	14088-1-AP	1:500
PPAR-γ	Proteintech	66369-1	1:1000
HSL	Bioworld	BS2742	1:500

p-HSL (phospho-S855)	Bioworld	BS4234	1:500
ATGL	Bioworld	BS7989	1:1000
CPT1α	Bioworld	BS70732	1:1000
FABP1	Bioworld	BS7533	1:1000
CD36	Bioworld	BS7861	1:1000
Tubulin-α	Bioworld	BS1699	1:10000
Secondary antibody			
Goat anti-Mouse IgG (H+L) -HRP	Bioworld	BS12478	1:10000
Goat anti-Rabbit IgG (H+L)-HRP	Bioworld	BS13278	1:10000
Rabbit Anti-Goat IgG (H+L)-HRP	Bioworld	BS30503	1:10000

Supplementary Table S3. miRNA and the corresponding primer sequences

Names	Sequence (5'to 3')	miRbase Accession
miR-3548	CAGCACTGTCCGGTAAGATGCC	MIMAT0017806
miR-182	TTTGGCAATGGTAGAACTCACACCG	MIMAT0005300
miR-27a-3p	TTCACAGTGGCTAAGTTCCGC	MIMAT0000799
U6	GGCAAGGATGACACGCAAAT	N/A

Figure captions

Figure S1. Volcano plot depicting differential expression of hepatic miRNAs in rats fed on α-lipoic acid. Each dot represents a miRNA. Green dots represent the significantly down-regulated miRNAs, whereas red dots show the markedly up-regulated miRNAs. Blue dots represent miRNAs that were not differentially expressed. Eight up-regulated miRNAs and eleven down-regulated miRNAs are revealed, in which 3 up-regulated miRNAs (miR-182, miR-27a-3p and miR-3548) are predicted to target FASN. None of up-regulated miRNAs is predicted to target FABP1.

Figure S1

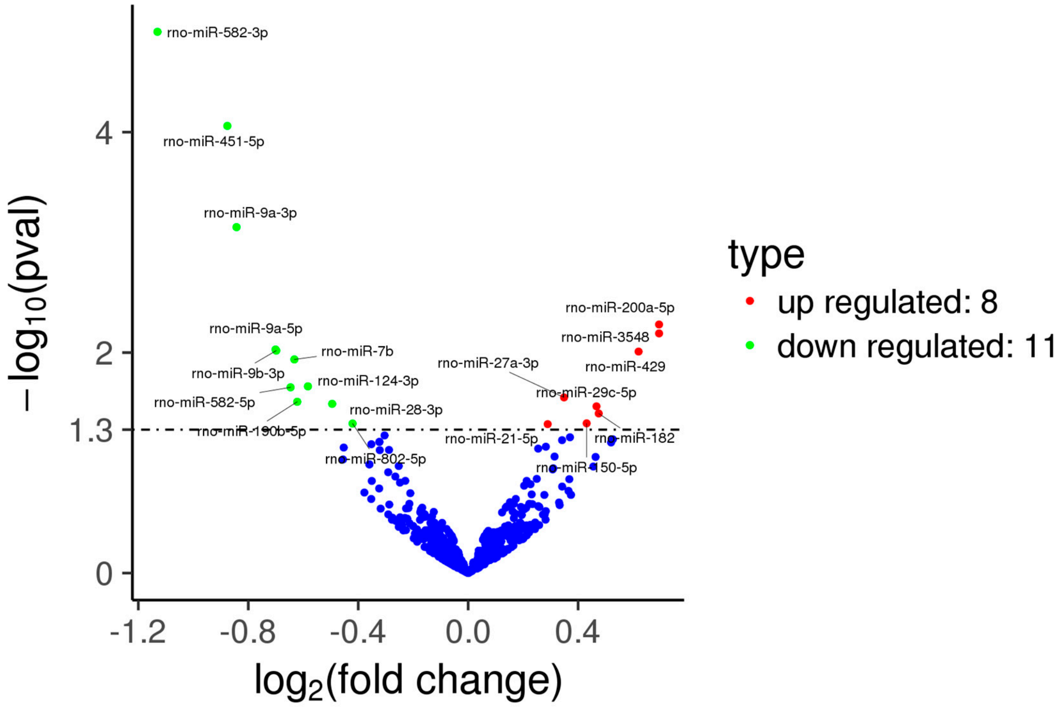
rno-miR-582-3p
rno-miR-451-5p
rno-miR-9a-3p
rno-miR-9a-5p
rno-miR-7b
rno-miR-9b-3p
rno-miR-124-3p
rno-miR-582-5p
rno-miR-28-3p
rno-miR-190b-5p
rno-miR-802-5p
rno-miR-200a-5p
rno-miR-3548
rno-miR-429
rno-miR-27a-3p
rno-miR-29c-5p
rno-miR-21-5p
rno-miR-182
rno-miR-150-5p
4
2
1.3
0
−log10(pval)
-1.2
-0.8
-0.4
0.0
0.4
log2(fold change)
type
up regulated: 8
down regulated: 11
